# Comparison between FlowTrac and Pulmonary Arterial Catheter in Off-Pump Cardiac Surgery Patients: “Why Did We Miss Our Appointment?”. Comment on Oh et al. Comparison between Fourth-Generation FloTrac/Vigileo System and Continuous Thermodilution Technique for Cardiac Output Estimation after Time Adjustment during Off-Pump Coronary Artery Bypass Graft Surgery: A Retrospective Cohort Study. *J. Clin. Med.* 2022, *11*, 6093

**DOI:** 10.3390/jcm12062343

**Published:** 2023-03-17

**Authors:** Luigi Vetrugno, Federico Barbariol, Cristian Deana

**Affiliations:** 1 Department of Medical, Oral and Biotechnological Sciences, University of Chieti-Pescara, 66100 Chieti, Italy; 2 Department of Anesthesiology, Critical Care Medicine and Emergency, SS. Annunziata Hospital, Via Dei Vestini 33, 66100 Chieti, Italy; 3 Anesthesia and Intensive Care 1, Department of Anesthesia and Intensive Care, Health Integrated Agency of Friuli Centrale, 33100 Udine, Italy

We read with great interest the study by Chahyun Oh et al., which compared estimates of cardiac output (CO) provided by the FloTrac system (CO-FloTrac) with those obtained with continuous thermodilution (COcont) after time adjustments using continuous recordings of intraoperative physiological data [1].

The study focused on cardiac surgery in a homogeneous group of 30 patients undergoing off-pump coronary artery bypass. Two patients were on hemodialysis, and two had a left ventricular ejection fraction of less than 40%.

The authors show the precision of CO-FloTrac to be clinically unacceptable, with a percentage error of 66.1%, as well as poor trending ability.

The Bland–Altman analysis showed a mean bias for COcont—CO-FloTrac of −0.94 (95% CI, −1.35 to −0.52) L/min, and the limits of agreement were −3.64 (95% CI, −4.44 to −3.08) L/min and 1.77 (95% CI, 1.21 to 2.57) L/min, respectively. In the discussion, the authors state that to the best of their knowledge, no study had previously compared continuous CO monitoring techniques in the context of cardiac surgery over the entire operative period.

We wish to respectfully inform the authors that over ten years ago, we published an article on uncalibrated arterial pulse cardiac output measurements in patients with moderately abnormal left ventricular function to evaluate the accuracy and precision of the Vigileo/FloTrac system (Edwards Lifesciences, Irvine, CA, USA) [2]. Albeit in a slightly different setting, our study compared intermittent cardiac output and continuous cardiac output measurements obtained from 20 patients using both pulmonary arterial catheters (PAC) and a Vigileo/FloTrac sensor device with 2nd generation software. We found only mild intraoperative and postoperative agreement between the estimates obtained with the Vigileo/FloTrac system vs. PAC.

We would like to comment on Chahyun Oh et al.’s findings using a metaphor [2]. When comparing two techniques for estimating cardiac output, the accuracy (compared against gold standard measurements) and precision (i.e., repeatability/reliability?) of each technique should also be considered in relation to the operative time. For example, if I want to meet a friend for a coffee in a new location, I must check that the information I have sent about both where and when is correct. Otherwise, we might miss each other by arriving in the wrong place or at the wrong time. Only when we both arrive in the right place at the right time can I conclude to have shared the information correctly.

Many mini-invasive devices, like the FloTrac CO monitoring tool, are precise (i.e., high repeatability) but insufficiently accurate (as indicated by the percentage error), with the potential for the latter to be also influenced by the surgical time. For example, while the reliability of FloTrac may be adequate after induction, its precision might become compromised after chest opening. Moreover, the surgical maneuvering of the heart whilst the pulmonary artery catheter (PAC) remains fixed could lead to a change in the position of the thermistor. So, in relation to our metaphor, comparing Flow-Trac and PAC across different surgical phases might lead to inaccuracies/inconsistencies in the comparison itself and, therefore, “the appointment being missed!”.

Furthermore, the PAC system should not be considered a gold standard for cardiac output monitoring [3]. Indeed, it is important that the precision of a reference method is first ascertained before using it for comparison with a second, as the poor reproducibility of measurements obtained with the former would limit the possibility of achieving good agreement with a second technique [4].

It was on the basis of such reasoning that Critchley and Critchley suggested a percentage error of 30% or less to indicate an acceptable level of agreement between two measurement techniques when, and only when, both the reference method(s) and the method(s) under assessment have a precision of at least 20% [5].

The pulmonary artery catheter is generally considered the clinical gold standard for measuring CO accuracy as well as the precision of other hemodynamic monitoring devices. However, the percentage error of PAC, as compared with the aortic flow probe (the true gold standard in cardiac output measurement), was shown to be greater than 40% [6], suggesting that the precision of PAC is actually less than the 20% threshold proposed by Critchley and Critchley [4]. Based on these findings, Payet et al. suggested that a percentage error of 45% should be accepted in clinical practice instead of the 30% previously proposed [6].

Observing the results of the Bland–Altman analysis presented in Figure 5 of Chahyun Oh et al., it would seem that the greatest dispersion in the accuracy of the measurements occurs for high values of CO, typically those >5 L/min, confirming previously reported data in the literature [7].

Therefore, we invite the authors to report the percentage error for all surgical time intervals because different cardiac surgical phases, e.g., the closed- vs. open-chest condition, could impact the overall measurements. Furthermore, we kindly ask them to comment on the possible modifications induced by arteriovenous fistula (if present in the two patients on hemodialysis) on FloTrac readings, as well as in the two patients whose EF values were <40%.
